# Tunable Self-Assembled Peptide Hydrogel Sensor for Pharma Cold Supply Chain

**DOI:** 10.1021/acsami.2c17609

**Published:** 2022-12-08

**Authors:** Tatiana N. Tikhonova, Dana Cohen-Gerassi, Zohar A. Arnon, Yuri Efremov, Peter Timashev, Lihi Adler-Abramovich, Evgeny A. Shirshin

**Affiliations:** † Department of Physics, M.V. Lomonosov Moscow State University, Leninskie gory 1/2, Moscow119991, Russia; ‡ SBIH Vorohobov’s City Clinical Hospital No. 67 MHD Moscow, 2/44 Salam Adil St., Moscow123423, Russia; § Department of Oral Biology, The Goldschleger School of Dental Medicine, Sackler Faculty of Medicine, The Center for Nanoscience and Nanotechnology, The Center for the Physics and Chemistry of Living Systems, Tel Aviv University, Tel Aviv69978, Israel; ∥ World-Class Research Center “Digital Biodesign and Personalized Healthcare”, Sechenov First Moscow State Medical University 8-2, Trubetskaya St., Moscow119991, Russia; ⊥ Institute for Regenerative Medicine, Sechenov University, 8-2 Trubetskaya St., Moscow119991, Russia

**Keywords:** defrost sensor, hydrogel, peptide self-assembly, pharma cold chain, fibrillation

## Abstract

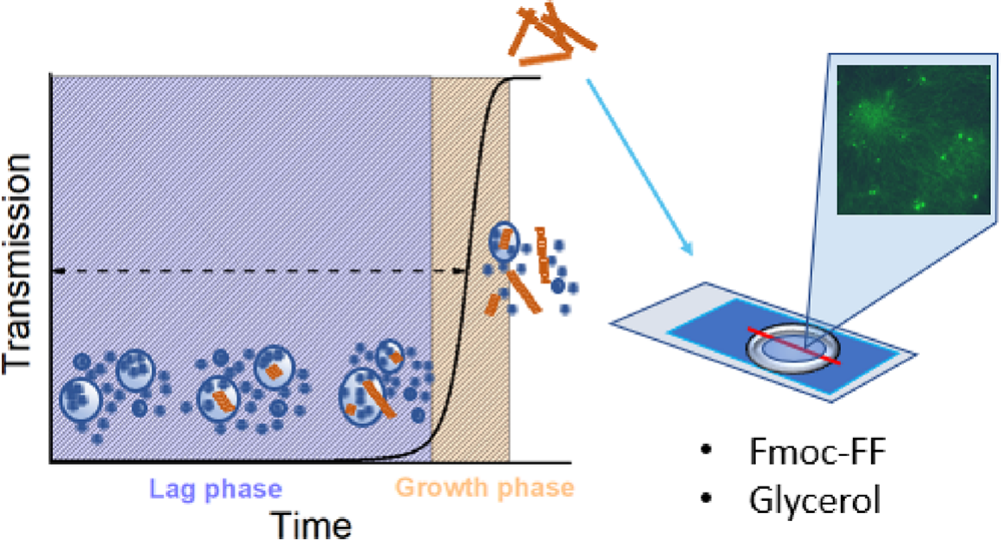

Defrost sensors are a crucial element for proper functioning of the pharmaceutical cold chain. In this paper, the self-assembled peptide-based hydrogels were used to construct a sensitive defrost sensor for the transportation and storage of medications and biomaterials. The turbidity of the peptide hydrogel was employed as a marker of the temperature regime. The gelation kinetics under different conditions was studied to detect various stages of hydrogel structural transitions aimed at tuning the system properties. The developed sensor can be stored at room temperature for a long period, irreversibly indicates whether the product has been thawed, and can be adjusted to a specific temperature range and detection time.

## Introduction

Self-assembly of short peptides has been extensively utilized for the development of a broad spectrum of biocompatible materials.^1−4^ Depending on the conditions, nano- and microstructures with different morphologies—monolayers, fibers, tubes, ribbons, vesicles, *etc*.—can be engineered by tuning the self-assembly pathway without special cross-linking.^5−8^ On the macro level, peptide-based hydrogels are three-dimensional materials used as scaffolds for regenerative medicine and for encapsulation or drug delivery systems.^9−13^ The sensitivity of peptide hydrogels to external factors makes them promising candidates for sensing applications.^14−16^ Importantly, the optical properties of peptide-based hydrogels during hydrogelation exhibit dramatic changes and strongly depend on the self-assembly kinetics and morphology of the formed nanostructures.^17,18^

**Figure 1:**
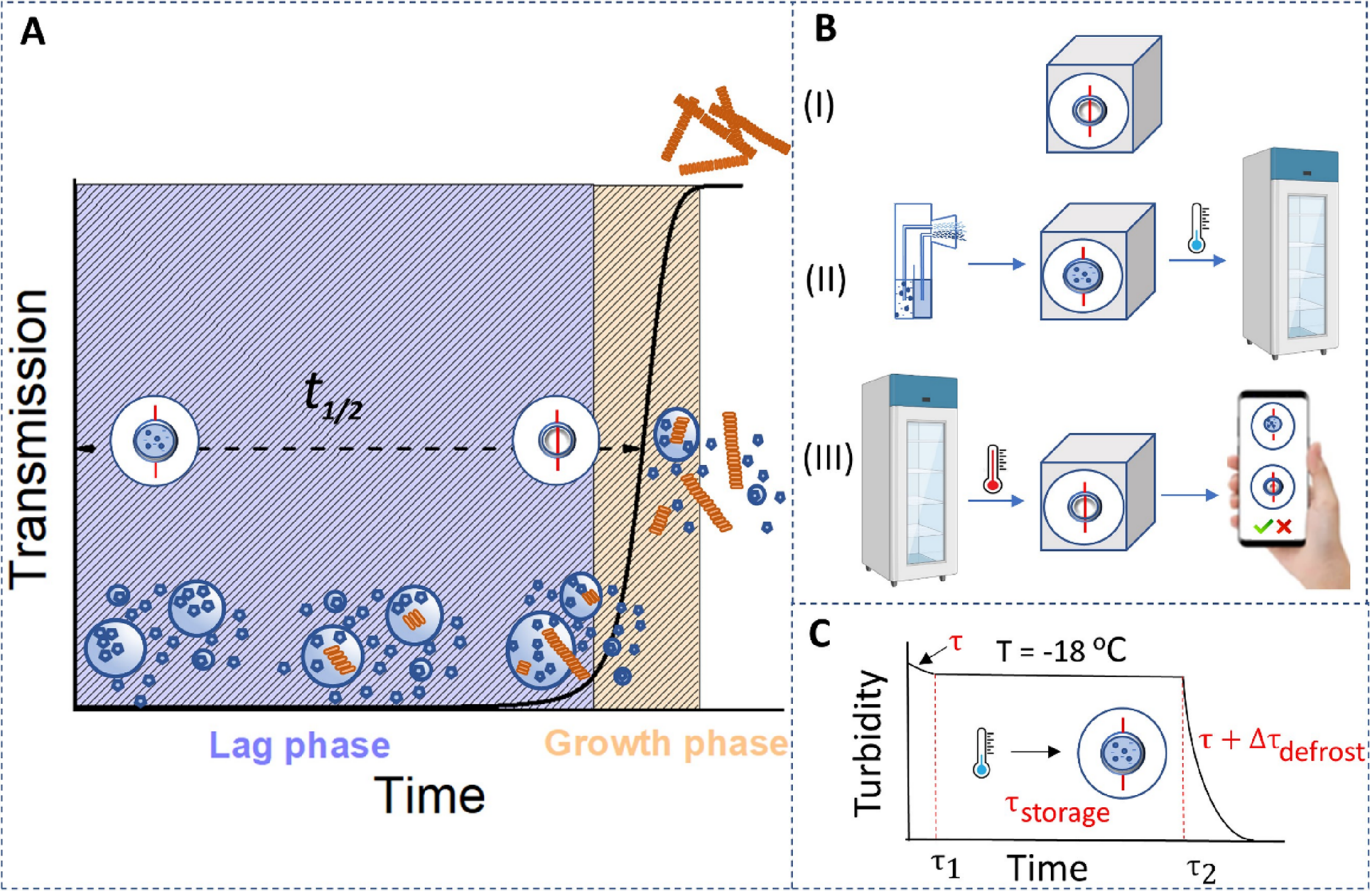
Schematic illustration of the peptide-based cold supply chain sensor. (A) Schematic illustration of the Fmoc-FF peptide hydrogelation process tracked via transmission. Lag phase: following the formation of metastable particles from peptide monomers in the supersaturated solution, the nucleation and growth of assemblies within the particles occur. Growth phase: the extension of fibers into the solution and the spheres-to-fibers transition lead to an abrupt turbidity decrease. (B) Schematic representation of the sensor activation. First, the sensor is placed above the controlled object (I), which is then stored in the refrigerator (II). After defrosting, the visual appearance of the sensor changes irreversibly (III) so that violation of the storage conditions is readily detectable by visual inspection. (C) Scheme of the defrost sensor operation mode. Upon defrosting, the sensor changes its appearance from turbid to transparent, and the duration of this transition is determined by the self-assembly time τ. The duration of the turbid appearance of the sensor at low temperatures (storage time, τ_storage_) can be tuned by changing the system’s parameters. The turbid-to-transparent state transition during defrosting is slightly slower compared to that at room temperature because of the additional time needed for the sensor to warm up.

Aromatic moieties such as fluorenylmethoxycarbonyl (Fmoc) can promote the self-assembly of peptides into a hydrogel in aqueous environments, as the aromatic group contribution includes both π–π stacking and hydrophobic interactions.^1^ Self-assembly of these peptides takes place because of the multiple noncovalent interactions, which allow the monomeric building blocks to self-assemble into ordered fibrous structures that later entangle and interact with one another to form the three-dimensional hydrogel matrix. The archetypal process of peptide hydrogelation includes several structural transitions, which can be described by the two-step nucleation model (Figure 1A).^19,20^ First, after the solvent switch, *i.e.*, dissolving the hydrophobic monomers in an organic solvent such as DMSO followed by dilution in water, amorphous spherical aggregates are formed. Subsequently, the aggregates may increase in diameter by incorporation of free peptide monomers from the solution depending on the peptide’s solubility and its free energy in the solution and in the aggregated phase. Simultaneously, nucleation within spherical assemblies takes place, and fibril-like structures start to form. At some point, the fibers’ length reaches the diameter of the spherical aggregate, and the elongation phase begins, as the spheres-to-fibers transition results in the formation of fibrillar hydrogels. The most pronounced change of the system’s optical properties is the transition from the turbid suspension to the transparent state of the fibrillar hydrogel, which is manifested as the growth phase in the kinetics of hydrogelation (Figure 1A). The kinetic pathway of self-assembly, the duration of different stages, as well as the morphology of the formed structures can be tuned by external factors such as temperature, ionic strength, pH, solvent, and the addition of other molecules.^21−24^ Hence, the turbidity and visual appearance of the system can be dynamically modulated and serve as an indicator of processes that influence the self-assembly, thus potentially serving as a basis for sensing.

Numerous medical products such as vaccines, reagents, medications, and biomaterials (e.g., blood plasma) must be stored at low temperatures to preserve their properties. Violation of the storage conditions can lead to a loss of their properties and safety.^25,26^ Monitoring the temperature of drugs and vaccines is a crucial element of cold chain logistics control in the pharmaceutical industry. The increased demand for cold chain logistics due to the crisis of COVID-19 pandemic raised the need for simple yet accurate sensors. Hence, a sensor changing its properties and, specifically, its visual appearance in a nonreversible manner upon defrosting is required. In this work, we developed a sensor with tunable parameters based on the self-assembly of the *N*-fluorenylmethoxycarbonyl-diphenylalanine (Fmoc-FF) peptide.^27,28^ The turbidity of the self-assembled peptide hydrogels is employed to construct a sensitive defrost sensor with a tunable working temperature range and alarm time for continuous monitoring of the cold chain logistics in the pharmaceutical industry.

## Results and Discussion

### Sensor Design

The sensor is designed as follows: a sticker with a red label (or QR code) is placed on the product (see Figure 1B(I)). Then, the sensor is activated by introducing a turbid peptide suspension onto the surface. The product with the sensor is then stored at a storage temperature *T*_storage_ (low temperature), and during the storage time τ_storage_, the sensor remains turbid and the label is visually concealed (see Figure 1B(II)). In the case of defrost, the peptide undergoes self-assembly, resulting in a transition to a transparent state after a certain alarm time, τ + τ_defrost_ (where τ is the time required for the peptide self-assembly and τ_defrost_ is the time required for the sensor defrost), which should be tunable. Repetitive freezing of the sample or any other manipulations should not return the sensor to its turbid state. Changes in the sensor state should be easily observed with a naked eye or with a smartphone (see Figure 1B(III)).

### Tuning of the Defrost Sensor Properties

**Figure 2:**
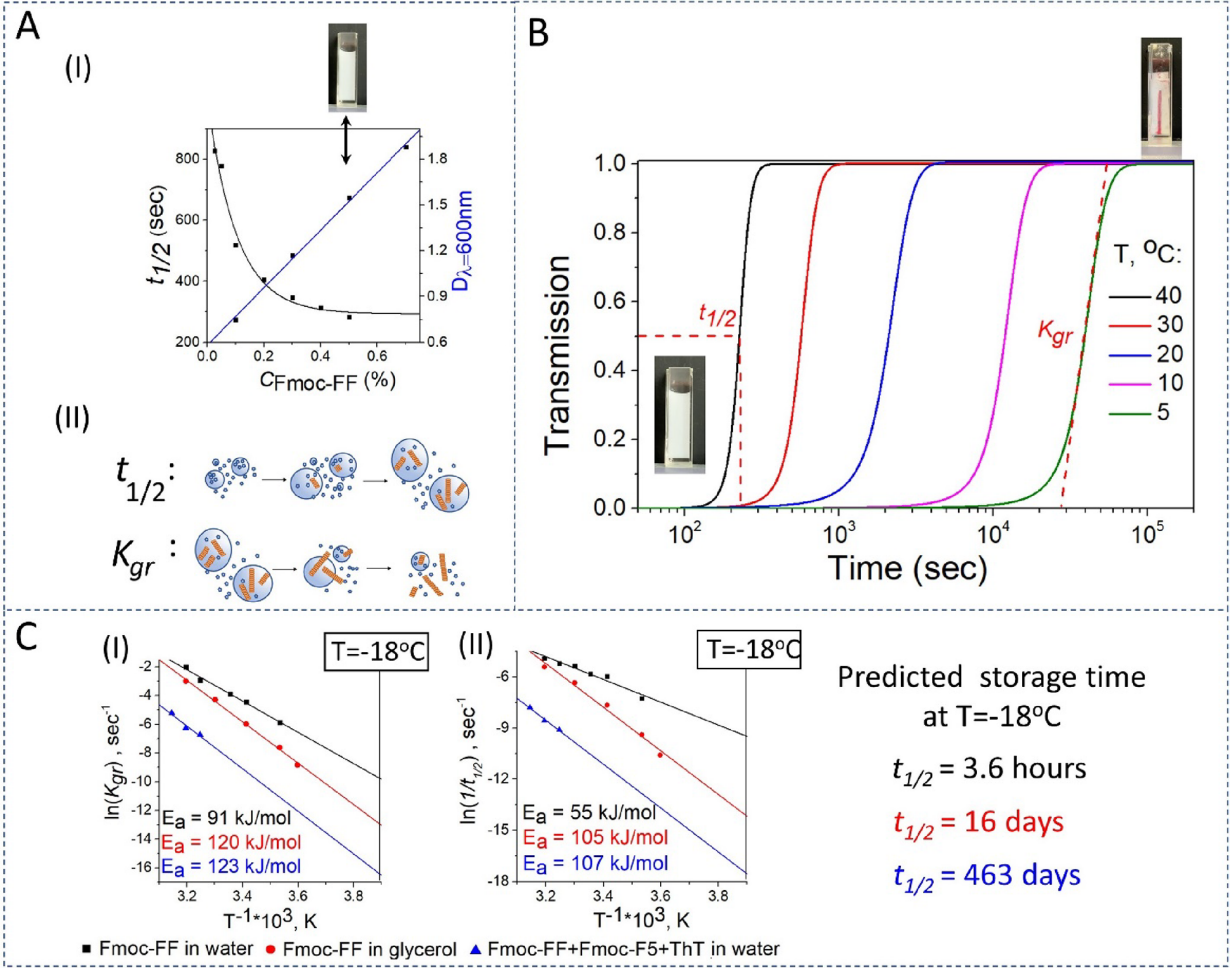
Tuning of the defrost sensor properties. (A) (I) The dependences of *t*_1/2_ obtained for the Fmoc-FF hydrogel formation in water (black line) and OD_600_ (blue line) on the peptide concentration. Inset: an image of the hydrogel at an Fmoc-FF concentration of 0.5%.(II) Schematic representation of the processes determining the *t*_1/2_ (lag-phase duration) and *K*_gr_ (growth rate) parameters. (B) Temperature dependence of the gelation kinetics (as monitored by light transmission). Lower temperatures are characterized by a longer lag phase and slower growth rate. Insets: the images of the turbid peptide suspension at the beginning and at the end of the gelation process. (C) Arrhenius plots for (I) the growth rate *K*_gr_ and (II) the 1/*t*_1/2_, where *t*_1/2_ is the lag-phase duration determined as the time at the level of 0.5 normalized transmissions as displayed in panel C, obtained for Fmoc-FF in water (black line), Fmoc-FF in glycerol, *C*_Gly_ = 70% (red line) and Fmoc-FF + Fmoc-^F5^F + Thioflavin T in glycerol, *C*_Fmoc-FF+Fmoc-F5F_ = 0.5%, *C*_ThT_ = 10 μM, *C*_Gly_ = 70% (blue line) systems.

The first challenge in the development of the defrost sensor based on the Fmoc-FF peptide self-assembly was to optimize its visual properties, *i.e.*, to make the defrost-induced optical switch observable by the naked eye. The peptide-based sensor is based on its turbidity in the initial state versus its transparency after gelation, which is inhibited by decreasing the temperature of the solution. Hence, the optical density (OD) of the system at the initial state must be ∼1.5 (∼30-fold light attenuation) to make it look “white” and opaque due to Mie scattering. For 2 mm thickness, obtaining such an OD requires a minimal Fmoc-FF concentration of 0.5% (blue line, Figure 2A(I)).

The increase in peptide concentration accelerates the hydrogel formation. Figure 1A(I) (black line) demonstrates that the hydrogelation time (obtained from the kinetic curves, Figure S1) decreased with the peptide concentration. Thus, optimization of the hydrogelation time *t*_1/2_ was required. This parameter is critical, as it determines how long the system stays turbid at low temperatures. For instance, if hydrogelation continues while the system is refrigerated, the sensor would become transparent after some time, although it would take a long time, and its function would be compromised. Although preparing the system in water would prevent the self-assembly process by ice formation, a frozen system—and even a frozen transparent hydrogel—looks turbid, thus hindering visual separation between the refrozen system (Figure S2D) and a frozen turbid suspension (Figure S2B). Consequently, to avoid the freezing of the system at the storage temperature *T*_storage_, we used water–glycerol mixtures.

The increase of glycerol concentration significantly elevates the viscosity.^29^ The changing glycerol concentration from 0 to 70% raises the viscosity ∼20-fold at room temperature. The increased viscosity leads to deceleration of all the diffusion-limited processes that accompany hydrogel formation.

The effect of the glycerol concentration ranging from 40 to 75% on the hydrogelation time is illustrated in Figure S3. When 0.5% Fmoc-FF was mixed with water, the hydrogelation time at room temperature, *T* = 23 °C, was ∼8 min. The addition of glycerol resulted in prolonged hydrogelation time, reaching 30 min at 75% glycerol (Figure S3). Importantly, water–glycerol solutions exhibit low freezing temperatures; for instance, for *C*_Gly_ = 70% glycerol, the freezing temperature is −40 °C.^30^ Hence, the transparent gel would not become turbid upon freezing, thus making it possible to immediately detect defrosting.

The possibility of tuning the sensor storage time was investigated by measuring the hydrogelation kinetics in the temperature range from 5 to 40 °C. The data were then fitted to the Arrhenius law and extrapolated to low temperatures. We observed that the activation energy for the lag phase and the growth rate increased with the glycerol concentration. The processes that correspond to the *t*_1/2_ (lag-phase duration) and *K*_gr_ (growth rate constant) parameters are shown in Figure 2A: 1/*t*_1/2_ is associated with the stage that starts with the nucleation inside the spheres until the size of the assemblies reaches the critical value. *K*_gr_ is associated with the process that starts when the protofibrils begin to escape from spheres, and the spheres-to-particles transition occurs. The increase of the activation energies for both the *K*_gr_ and 1/*t*_1/2_ means that glycerol influences all the self-assembly stages from nucleation inside the spheres to fibril elongation, although its influence on the initial stage is more pronounced, as increasing the glycerol concentration to 70% led to a dramatic twofold increase of the activation energy for 1/*t*_1/2_ (*E*_a_ = 55 kJ/mol for water versus *E*_a_ = 105 kJ/mol for 70% glycerol). The values of activation energies obtained for the Fmoc-FF system in different environments are presented in Table 1.

**Table 1:** The Values of Activation Energy *E*_a_ in kJ/mol for the growth rate (*K*_gr_) and Lag-Phase Duration (1/*t*_1/2_) for the Fmoc-FF system in different environments: (a) Fmoc-FF in Water, *C*_Fmoc-FF_ = 0.5%; (b) Fmoc-FF in Glycerol, *C*_Fmoc-FF_ = 0.5%, *C*_Gly_ = 70%; and (c) Fmoc-FF + Fmoc-^F5^F + ThT in Glycerol, *C*_Fmoc-FF+Fmoc-F5F_ = 0.5%, *C*_ThT_ = 50 μM, *C*_Gly_ = 70%

	Fmoc-FF in water [kJ/mol]	Fmoc-FF in glycerol [kJ/mol]	Fmoc-FF + Fmoc-^F5^F + ThT in glycerol [kJ/mol]
1/*t*_1/2_	55	105	107
*K*_gr_	91	120	123

According to the estimated value of the activation energy, at −18 °C, 0.5% Fmoc-FF in 70% glycerol would start becoming transparent after 16 days and would become completely transparent after 32 days. The theoretical predictions coincided with the experiments. Hence, we tested the sensor with the abovementioned parameters by studying its behavior when stored at −18 °C.

It should be noted that there are other ways to decelerate the hydrogelation kinetics: for instance, the presence of the fluorescence dye Thioflavin T may result in a 10-fold increase of the hydrogelation time.^17^ Also, the mechanical and kinetic properties of the peptide hydrogel can be synergistically modulated by a multicomponent assembly, e.g., using a 1:1 ratio of Fmoc-FF and Fmoc-pentafluoro-phenylalanine (Fmoc-^F5^F).^31^ The use of such mixtures also decreases the rate of gelation and can be applied for tuning the parameters of the defrost sensor. Using the Arrhenius law, we have tested how long Fmoc-FF + Fmoc-^F5^F + ThT in the glycerol system would stay turbid when stored at −18 °C.

Arrhenius plots for (I) the growth rate *K*_gr_ and (II) the 1/*t*_1/2_ obtained for Fmoc-FF + Fmoc-^F5^F + ThT in glycerol can be seen in Figure 2C (blue line). According to the estimated value of the activation energy, at room temperature, this system would start becoming transparent after 11 h, whereas at −18 °C, it would become transparent only after 463 days; hence, such a system could be used for long-term tracking of the pharma cold chain. It should be mentioned that the Fmoc-FF + Fmoc-^F5^F + ThT in the glycerol system was tested: it was put in the refrigerator at −18 °C for 6 months, and it stayed turbid. If we compare the two systems that can be used as active media for long-term sensors, (1) Fmoc-FF in glycerol and (2) Fmoc-FF + Fmoc-^F5^F + ThT in glycerol, it should be mentioned that both of them have advantages and disadvantages. Although the Fmoc-FF + glycerol system becomes transparent much earlier in comparison with Fmoc-FF + Fmoc-^F5^F + ThT in the glycerol system and the utility of the sensor is questionable if it is only stable up to 16 days of monitoring, whereas the Fmoc-FF + Fmoc-^F5^F + ThT in the glycerol system becomes transparent only after 463 days (as predicted by the Arrhenius plot), for the Fmoc-FF + glycerol system, the sample defrost response time is very suitable, namely, 25–30 min, whereas this parameter for Fmoc-FF + Fmoc-^F5^F + ThT in the glycerol system is much higher. Determining the most suitable active media sensor parameters will be the subject of future research.

### Hydrogelation in the Presence of Glycerol: Insights from Microscopy

The mechanism of hydrogel formation deceleration may vary depending of the substances presented in the self-assembly system: for instance, in the case of fluorescence dye Thioflavin T, its presence decreases the interaction energy between the stacked peptides, thus leading to longer lag times and lower growth rates.^17^ The presence of the peptide mixture Fmoc-FF + Fmoc-^F5^F also decreases the hydrogel formation kinetics, which is explained by the slow diffusion of the building blocks during the process of structural organization into fibers within a viscous solution, as the Fmoc-^F5^F solution became very viscous immediately following the dilution in water, whereas the Fmoc-FF solution remained in a liquid state for several minutes.^31^

**Figure 3:**
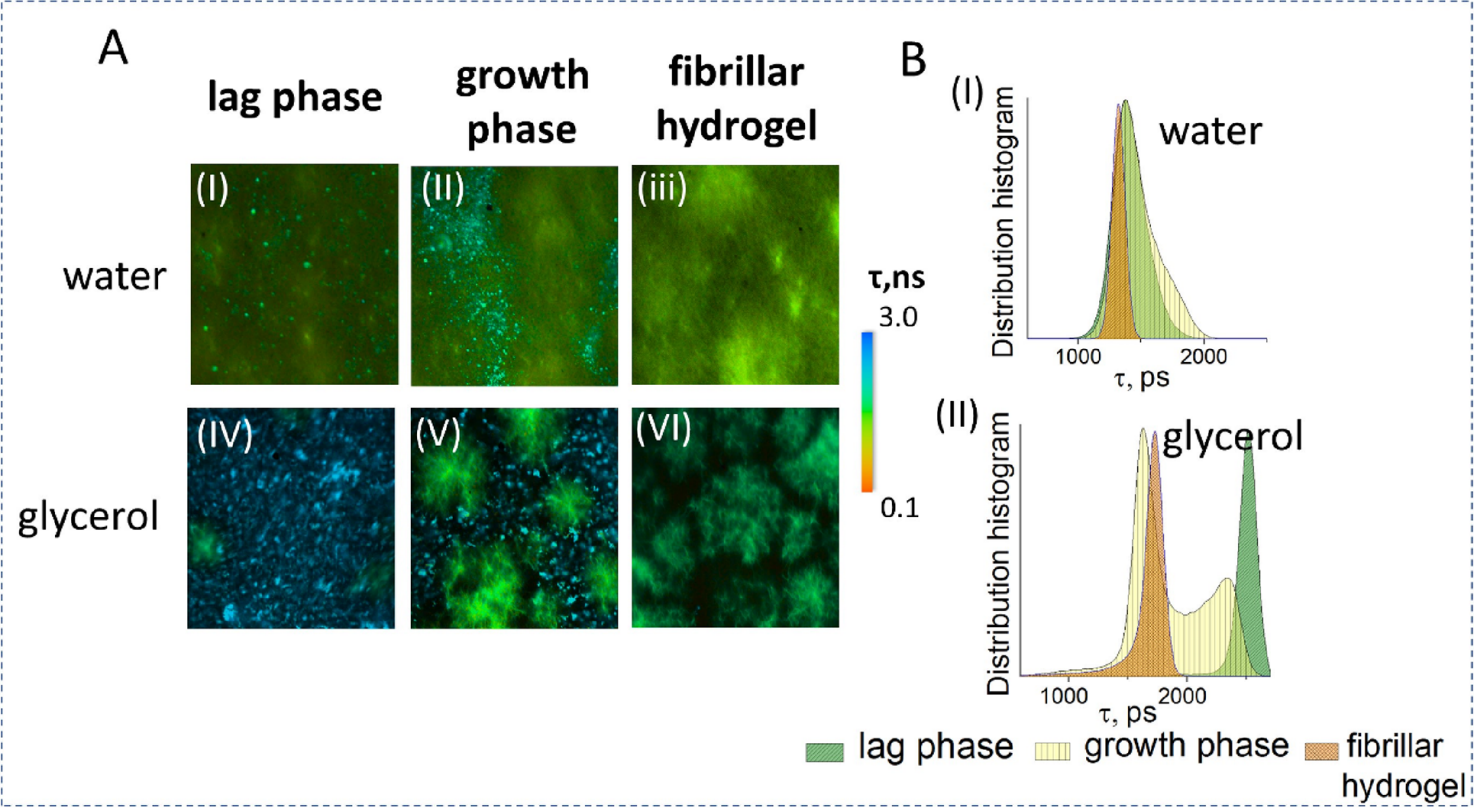
Microscopy analysis during the gelation process. (A) Fluorescence lifetime imaging microscopy of the representative stages of the hydrogel self-assembly obtained at 405 nm excitation for (I–III) Fmoc-FF in water and (IV–VI) Fmoc-FF in glycerol, *C*_Fmoc-FF_ = 0.5%, *C*_Gly_ = 70% with 10 μM Thioflavin T staining. (B) The Thioflavin T fluorescence lifetime distribution for hydrogel in different stages in Fmoc-FF (I) in water and (II) in the glycerol system is presented.

To explain why the presence of glycerol prolongs the sensor hydrogelation time, the structural changes in the Fmoc-FF in water and Fmoc-FF in glycerol were examined using fluorescence lifetime imaging microscopy (FLIM). Several stages could be identified during Fmoc-FF self-assembly in water as well as in glycerol using a low concentration of the ThT dye as a probe.^17^ First, amorphous spherical aggregates were formed (Figure 3(A(I)), see figures for water, lag phase). This process corresponds to the initial region of the sigmoidal curve of the system’s turbidity over time observed during hydrogel formation (Figure 2B). The growth phase corresponds to the process when the amorphous spherical aggregates increase in size and form larger spheres, and then the transition from spheres to fibers (fibrillar hydrogel stage) occurs. The increase of solution viscosity caused by the addition of glycerol influenced that particular stage of hydrogel formation: the addition of 70% glycerol slows down the process of spheres-to-fibers transition, clustering the big spheres together, so that mature fibrillar hydrogel forms later compared to the hydrogel formation in water (see Figure 3A(II)). It is also seen that the final stages for Fmoc-FF in water and glycerol systems have different structures: the aqueous Fmoc-FF hydrogel consists of homogeneously distributed fibers (Figure 3A(III)), whereas the Fmoc-FF in glycerol is characterized by the presence of “clouds” (Figure 3A(VI), fibrillar hydrogel for water and glycerol).

ThT is the molecular rotor molecule, whose fluorescence decay is determined by the viscosity of microenvironment.^32^ The evolution of the ThT fluorescence lifetime for Fmoc-FF in a glycerol system is shown in Figure 3B(II), where it can be seen that the ThT lifetime for the lag phase that corresponds to the presence of spherical particle is τ ∼ 2500 ps, and the fibrillar hydrogel is characterized by the τ ∼ 1730 ps, whereas the distribution for the growth phase where the transition from spheres-to-fibers occurred is bimodal, indicating incorporation of the ThT fluorescence probe into the structures with different microenvironments. No multimodal distribution is observed for the growth phase in Fmoc-FF in water, demonstrating that such system is more homogeneous (see Figure 3B(I)).

It can be suggested that the two main reasons behind such deceleration of hydrogel formation kinetic are (1) the increase of media viscosity that prevents all diffusion processes during gelation and (2) the stabilization effect of glycerol on peptides by the preferential accumulation of glycerol around Fmoc-FF that decreases the interactions of water molecules with hydrophobic peptides and thus slows down the aggregational processes that take place during hydrogel formation.

In our experiment, the concentration of glycerol was 70%, and the viscosity increased 20-fold at room temperature, so the diffusion of peptides and their aggregation slowed down strongly. For instance, in Matthews *et al*., the decreasing entropy and increasing bulk viscosity in the presence of glycerol were discussed as the main mechanisms for the unexpected formation of a microfibrillar gel in SDS and glycerol mixtures at a critical gelation concentration.^33^ Also, Kulmyrzaev *et al.* showed a decrease in protein aggregation rate and gelation that was suggested by the increase in the viscosity.^34^

On the other hand, glycerol is known to stabilize the native structure of proteins and peptides, thus changing their hydration and aggregation rate.^35,36^ To separate these two mechanisms on the hydrogelation kinetics, the following experiment was carried out. The Fmoc-FF hydrogelation was studied at different glycation concentrations and at two temperatures, *T* = 20 and 25 °C, and *C*_Gly_ was varied from 0 to 75%.

**Figure 4:**
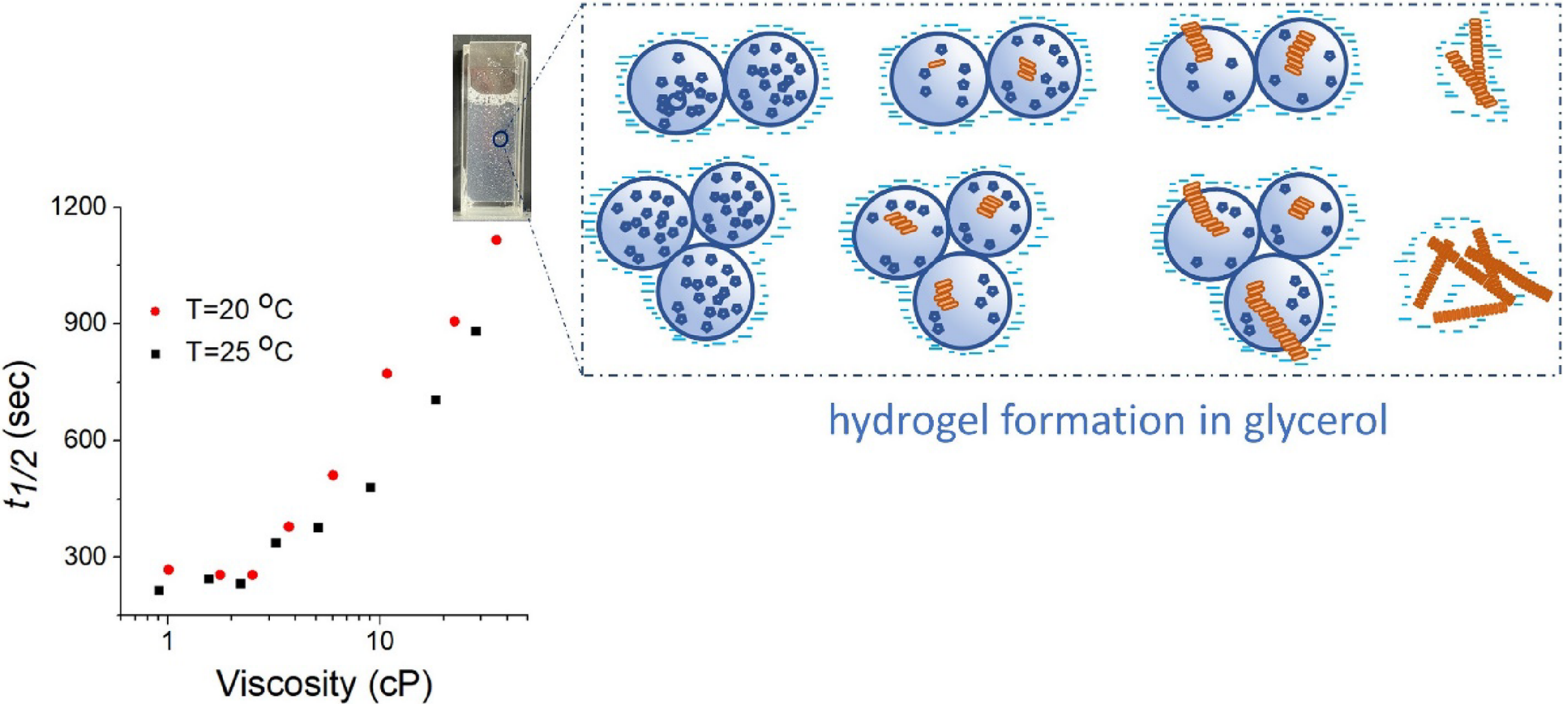
The Fmoc-FF hydrogelation process tracked via transmission at different glycation concentration. *C*_Gly_ was varied from 0 to 75%, which was translated into viscosity units. The dependence was obtained at *T* = 20 and 25 °C. The inset shows the photo of the mature hydrogel at high media viscosity (*C*_Gly_ was varied from 60 to 75%) and the scheme of hydrogel formation in glycerol.

Figure 4 demonstrates that the key role in the deceleration of hydrogel formation is due to viscosity alterations: at glycerol concentrations of *C*_Gly_ from 0 to 40%, no changes are observed for *t*_1/2_, whereas the consequent twofold increase of glycerol concentration, where viscosity changes, leads to the pronounced increase of the lag-phase duration. Hence, we consider that changes in the hydrogel formation rate and morphology are caused by viscosity-induced deceleration of the peptide diffusion-limited processes (Figure 4, inset). Immediately after dilution of the peptide stock solution in water, large spherical structures are observed that are surrounded by the “cloud” of glycerol that prevents its growth (see Figure 3A(V)). Next, the transition from spheres to fibers occurred, which was decelerated by limited diffusion caused by the increase in the viscosity. The mature fibrillar hydrogel consists of large clusters from fibrils, so the photo of the final hydrogel contains a great number of aggregates (see Figure 3A(VI)).

### Vibration Influence on the Hydrogel Formation

**Figure 5:**
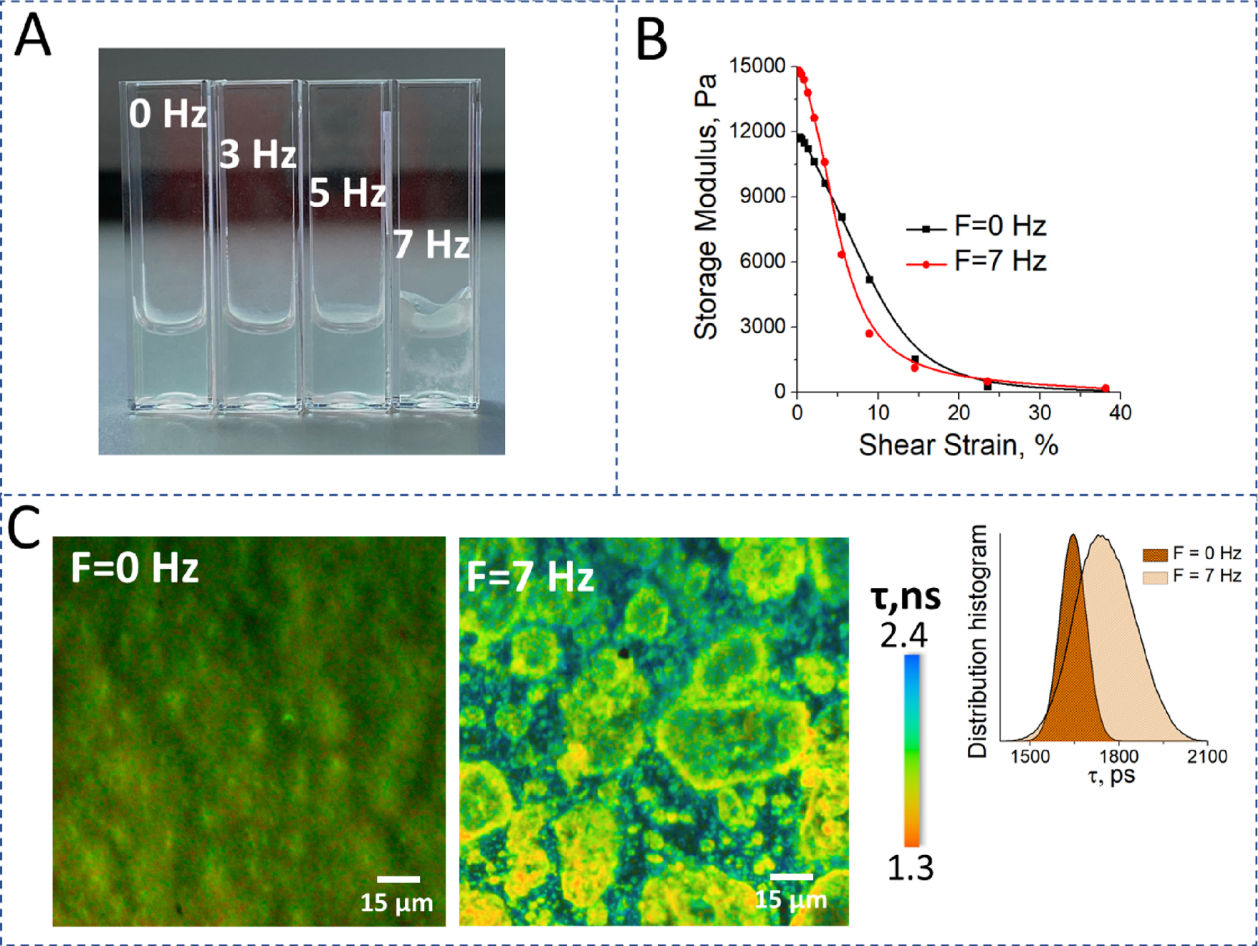
Vibration influence on the hydrogel formation. (A) The photos of the Fmoc-FF hydrogel formed at 0, 3, 5, and 7 Hz shaking frequency (from left to right). (B) Time-sweep oscillatory test for Fmoc-FF hydrogel for the control sample and for the sample that was shaken at *F* = 7 Hz for 10 min. (C) Fluorescence lifetime imaging microscopy images of the control sample (left) and the hydrogel that was formed at *F* = 7 Hz (right). The images were obtained at 405 nm excitation for the Fmoc-FF in water, *C*_Fmoc-FF_ = 0.5%, with 10 μM ThT. In the inset, the ThT fluorescence lifetime distribution is presented.

In addition, we also considered the effects of mechanical vibration and shear forces on hydrogel formation. For the cold chain application, it would be a major challenge to utilize such sensor if vibrations or shear affected the turbidity of the gel. For this purpose, three different frequencies were used: 3, 5, and 7 Hz. The kinetics of hydrogel formation was compared for the Fmoc-FF in water that was formed without shaking and for three samples where the hydrogel was formed during its shaking with 3 Hz (see Video S1), 5 Hz (see Video S2), or 7 Hz (see Video S3). As it can be seen from these videos, the shaking of the system at a 3 Hz frequency did not influence the rate of hydrogel formation; the 5 Hz frequency slightly slowed down the process of hydrogel formation, although the structure of the final hydrogel was unchanged, and application of 7 Hz led to the formation of the turbid final state. The system did not become transparent during shaking. Only when the shaking process was stopped did hydrogelation occur, although the morphology of the system was altered significantly compared to that of the control sample (see Figure 5A). To assess whether the observed changes in morphology were accompanied by changes in the mechanical properties of the Fmoc-FF hydrogel, rheological measurements were carried out. The dependence of the storage modulus *G*’ during application of shear strain for the control sample, *F* = 0 Hz (black line) and hydrogel that was formed at 7 Hz (red), is presented in Figure 5B. Although the rigidity of the sample that was formed at 7 Hz was higher, its structure was less stable: application of shear strain led to its earlier fracture compared to the control sample.

The obtained data were consistent with changes in the ThT fluorescence lifetime: the sample formed at 7 Hz and with a more rigid structure was characterized by slower ThT relaxation (1800 ps compared 1600 ps for the control sample). The fluorescence of ThT is sensitive to the polarity, temperature, and viscosity of the microenvironment^32^ and can be utilized as an indicator for alterations in the ThT binding sites accompanying hydrogelation.

To summarize, we observed that the hydrogelation process in the studied system is independent of the influence of vibrations, although at frequencies exceeding 5 Hz, the self-assembly process is partly disrupted. However, the obtained results indicated that the presence of low-frequency vibrations, which can be expected during the sample transportation, will not compromise the function of the developed sensor.

### Operation of the Defrost Sensor

**Figure 6:**
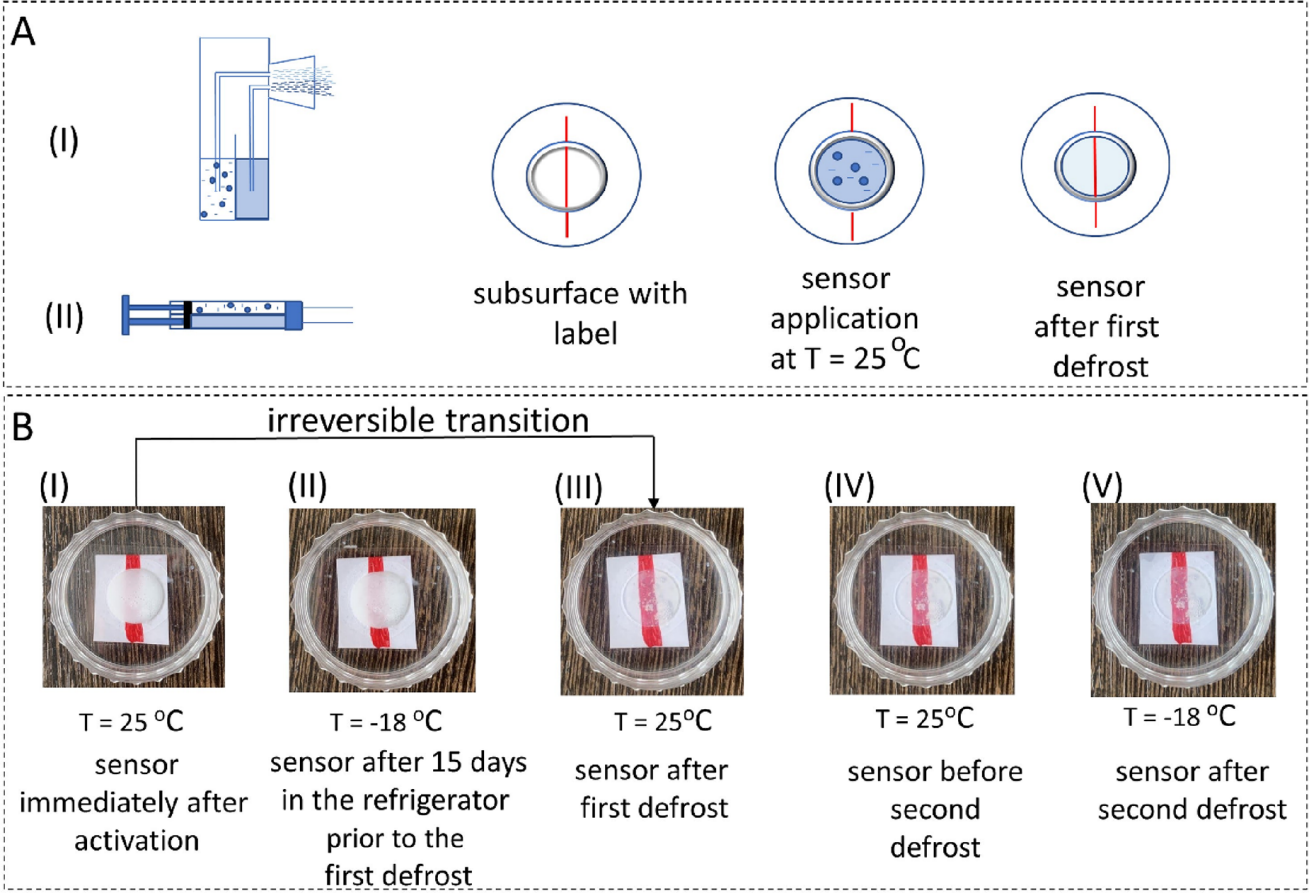
Deposition of the hydrogel-based sensor and its performance. (A) Schematic illustration of two different ways of coating the red-labeled sensor subsurface: (I) by airbrush and (II) by syringe. (B) Photos of a defrosted sensor at different stages of its functioning: (I) immediately after activation, *T* = 25 °C; (II) after 15 days in the refrigerator, *T* = −18 °C; (III) after the first defrost, *T* = 25 °C; (IV) before the second defrost, *T* = 25 °C; and (V) after the second defrost, *T* = −18 °C.

The next aim was the deposition of the hydrogel-based sensor. After the solvent switch, *i.e.*, upon dilution of the DMSO peptide stock solution into water, hydrogelation is immediately initiated. Hence, the components must be stored separately and mixed during the sensor deposition onto the controlled object. Two approaches were used to mix the DMSO stock solution of Fmoc-FF and the glycerol–water mixtures during deposition of the sensor (Figure 6A): (I) using an airbrush and (II) using connected syringes.

When the airbrush was applied for the deposition of the sensor over the red label, the two suspensions (peptide + DMSO and aqueous glycerol solution) were mixed simultaneously, and the mixture was uniformly distributed over the surface. A video of this process is available in the Supporting Information (Video S4). Another way of coating the red-labeled sensor subsurface was using two syringes with connected needles that allowed more precise application of the turbid solution to the subsurface (see Video S5).

After the mixed suspension was deposited on the surface, the red label became visually obscured (Figure 6B(I)). While the sensor was stored at -18 °C, it remained turbid for at least 16 days (Figure 6B(II)), as it was predicted from the activation energy-based estimations. Hence, the storage time calculated from the Arrhenius plot can be used to obtain a general prediction of how long the sensor of a certain composition would remain turbid in the freezer.

After the first defrost, the hydrogel became transparent within 25 min at room temperature, and this transition was irreversible (Figure 6B(III)). The *T*_1/2_ here exceeded the one obtained for the initially prepared system (8 min) as it also includes the time required for warming from −18 °C to room temperature. Then, the sensor was frozen again; yet the sensor remained transparent (red label is clearly visible) even at temperatures lower than the storage temperature (up to −40 °C, which is the freezing temperature for glycerol with *C*_Gly_ = 70%) and after the second defrost (Figure 6B(IV–V)). Hence, using the described parameters of the peptide sensor allowed detection of the defrost event after only 25 min of exposure to room temperature for at least 32 days of storage. We note that such a short storage time was intentionally selected for the laboratory tests, although, as shown in Figure 2C, it can be adjusted to years depending on the purpose.

## Conclusions

This work describes the fundamental research and analysis of utilizing self-organizing systems based on short peptides for biosensor applications, namely, to construct a defrost sensor for continuous monitoring of the pharma cold chain. The gelation kinetics under various conditions was analyzed, allowing the identification of individual stages of structural transitions and testing the models of gel formation, hence giving a possibility to develop new approaches to tune the system properties. We present a defrost sensor that possesses several important properties: (1) The sensor can be stored at room temperature for a long period. This effect was assessed by dividing the initial mixture components into two different vessels in such a way that the process of sensor activation starts immediately before it is placed into the freezer. (2) The sensor has a significant effect on activation. Thus, after coating the red-labeled subsurface using a specific Fmoc-FF peptide concentration, the label became invisible. (3) The sensor uniquely indicates that the sample has been defrosted, and it cannot be returned to its original state after defrosting. (4) The sensor can be adjusted to a specific temperature range to be used for long-term storage of samples in the freezer (i.e., no gelation process would be observed at low temperatures). For this purpose, the conditions for deceleration of gelation kinetics at low temperatures were obtained by using a defined glycerol concentration. The suggested peptide-based sensor paves the way for a tunable platform of pharma cold chain monitoring.

## Experimental Section

### Materials

The Fmoc-FF and Fmoc-^F5^F were purchased from GL Biochem (China). Thioflavin T (ThT) was obtained from Sigma-Aldrich (Germany). The glycerol was purchased from MP Biomedical (USA). The Fmoc-FF hydrogel was prepared using the solvent-switch method by diluting a dimethyl sulfoxide (DMSO) stock solution of Fmoc-FF with double distilled water to a final concentration of 0.5 wt % (9.5 mM); the final concentration of DMSO in the solution was 5%.^37^ This procedure resulted in the formation of a turbid Fmoc-FF suspension, which became more optically transparent after a certain time (depending on external conditions, such as temperature). The temperature in the experiments was controlled and set at 5 or 40 °C. The sensor was stored at −18 °C. To prevent the hydrogel from freezing in the freezer, the concentration of glycerol was used, *C*_Gly_ = 70%.

### Turbidity Measurements

Turbidity measurements were performed by measuring the transmission of a 633 nm diode laser through a cuvette (with a 2 mm optical path) with a Maya 2000 PRO (Ocean Optics, USA) spectrometer in a 180° geometry. The exposure time was set to 100 ms. The turbidity and time-resolved fluorescence measurements were carried out simultaneously (for the same sample). To obtain the Arrhenius plot, the turbidity measurements were carried out in the temperature range from 5 to 40 °C.

### Fluorescence Lifetime Imaging Microscopy (FLIM) Measurements

Fluorescence lifetime imaging microscopy (FLIM) measurements were performed using a MicroTime 200 STED microscope (PicoQuant GmBH, Germany) with a 405 nm laser as the excitation source. Measurements were carried out at a pulse rate of 40 MHz, a pulse duration of 40 ps, and a maximum power of 50 μW. The laser beam was focused on a sample with a 100 × 1.4 NA oil immersion objective (UplanSApo, Olympus, Japan). Fluorescence emission was detected using single photon-counting modules (Excelitas, USA). A long-pass emission filter (ET425Ip) with a cutoff wavelength at 425 nm was used to block the laser light from the detector. By setting the dwell time to 0.3 ms and for a pixel and a pixel size of 0.100 μm/px, the total image acquisition time was 70 s for an image size of 400 × 400 pixels, i.e., 80 × 80 μm. The measurements were carried out at 20 °C. For FLIM measurements, the concentration of Thioflavin T was *C*_ThT_ = 10 μM.

### Arrhenius Plot

In Figure 2C, the dependences of ln *K_gr_* on 1/*T* and ln 1/*t*_1/2_ on 1/*T* are presented, where *K* is the constant rate, *t*_1/2_ is time at level of 0.5 normalized turbidity, *T* is absolute temperature, K. *K*_gr_ = 1/dx, where dx is the slope of the curve that was determined from sigmoidal approximation of experimental data. The activation energies were determined from the following equation:
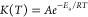
where *E*_a_ is the activation energy, *A* is the pre-exponential factor, and *R* is the gas constant.

### Vibration Influence Test

The experiment with the Fmoc-FF hydrogel formed at 0, 3, 5, and 7 Hz shaking frequency was carried out with an IKA MS 3 basic shaker (Germany). The corresponding amplitudes were 0, 2.3, 2.5, and 1.3 mm. The time-sweep oscillatory test for the Fmoc-FF hydrogel for the control sample and for the sample that was shaken at *F* = 7 Hz was measured using a Physica MCR 302 rheometer (Anton Paar GmbH, Graz, Austria) at 25 °C with a parallel plate geometry (25 mm in diameter).

## Supporting Information Available

Additional plots: the time course of turbidity for the Fmoc-FF system measured at 0.025–0.5% peptide concentration in water. Similar data were obtained for the glycerol solutions. Photos of a defrosted sensor at different stages of its use in water. *C*_Fmoc-FF_ = 0.5%. Images of hydrogel formation by 0.5% Fmoc-FF at different glycerol concentrations, as indicated (PDF)
Video S1: video of kinetics of hydrogel formation during its shaking with 3 Hz (MOV)
Video S2: video of kinetics of hydrogel formation during its shaking with 5 Hz (MOV)
Video S3: video of kinetics of hydrogel formation during its shaking with 7 Hz (MOV)
Video S4: video of coating the red-labeled sensor subsurface by airbrush (MOV)
Video S5: video of coating the red-labeled sensor subsurface by syringe (MOV)

## Supplementary Material

am2c17609_si_001.pdf

am2c17609_si_002.mov

am2c17609_si_003.mov

am2c17609_si_004.mov

am2c17609_si_005.mov

am2c17609_si_006.mov

## ABBREVIATIONS

Fmoc-FF: *N*-fluorenylmethoxycarbonyl diphenylalanine peptide
Fmoc-^F5^: Fmoc-pentafluoro-phenylalanine
DMSO: dimethyl sulfoxide
