# Supplementary material for: Tunable Self-Assembled Peptide Hydrogel Sensor for Pharma Cold Supply Chain
Source: ACS Appl Mater Interfaces. 2022 Dec 8;14(50):55392–401. doi: 10.1021/acsami.2c17609 (PMC9782340; doi:10.1021/acsami.2c17609)
Supplement: Supplementary file 1 — am2c17609_si_001.pdf [file am2c17609_si_001.pdf]

Supporting Information

Tuneable Self-assembled Peptide Hydrogel Sensor for Pharma Cold Supply Chain

Tatiana N. Tikhonova,[1],[2] *Dana Cohen-Gerassi,*[3] *Zohar A. Arnon,*[3] *Yuri Efremov,*[4],[5] *Peter Timashev,*[4],[5] *Lihi Adler-Abramovich,**[3] *Evgeny A. Shirshin**[1],[4]

[1] Department of Physics, M.V.Lomonosov Moscow State University, Leninskie gory 1/2, 119991, Moscow, Russia

[2] SBIH Vorohobov's City Clinical Hospital №67 MHD Moscow, 2/44 Salam Adil St., Moscow, 123423, Russia

[3] Department of Oral Biology, The Goldschleger School of Dental Medicine, Sackler Faculty of Medicine, The Center for Nanoscience and Nanotechnology, The Center for the Physics and Chemistry of Living Systems, Tel Aviv University, Tel Aviv, 69978, Israel

[4] World-Class Research Center "Digital biodesign and personalized healthcare", Sechenov First Moscow State Medical University 8-2, Trubetskaya st., Moscow, 119991, Russia

[5] Institute for Regenerative Medicine, Sechenov University, 8-2 Trubetskaya st., Moscow, 119991, Russia

Corresponding Authors:

Evgeny A. Shirshin, email: shirshin@lid.phys.msu.ru

Lihi Adler-Abramovich, email: lihiA@tauex.tau.ac.il

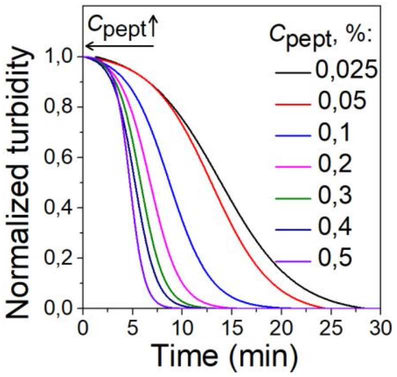

Figure S1. The time-course of turbidity for the Fmoc-FF system measured at 0.025-0.5% peptide concentration in water. Similar data was obtained for the glycerol solutions.

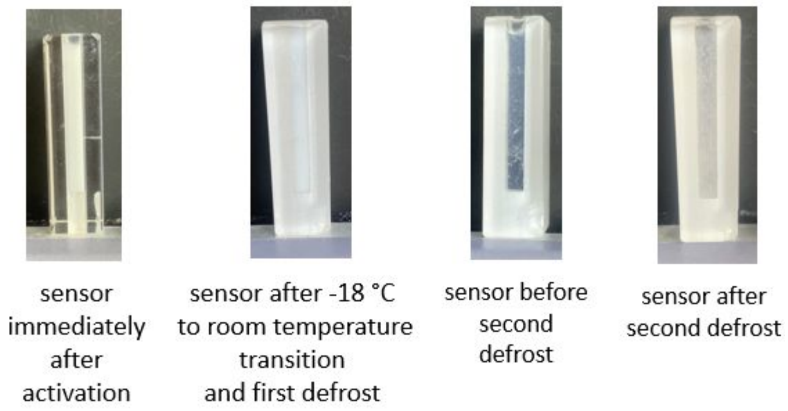

Figure S2. Photos of a defrosted sensor at different stages of its use in water. [Fmoc-FF] = 0.5%.

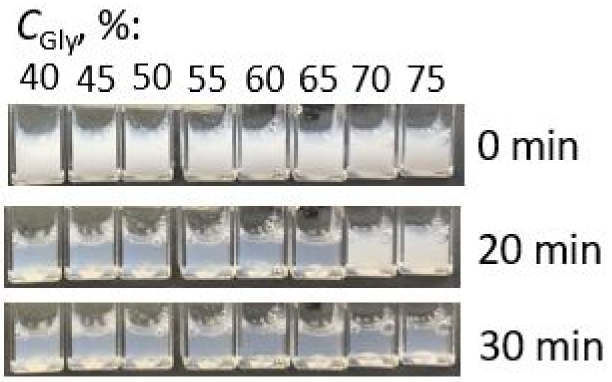

Figure S3. Images of hydrogel formation by 0.5% Fmoc-FF at different glycerol concentrations, as indicated.
